# Complete Obliteration of Urethra—Gangrene of Bladder

**Published:** 1842-07-16

**Authors:** 


*Complete Obliteration of the Urethra—Gangrene of the Bladder.*—M. Bosc presented to the Anatomical Society the urinary organs of a man in whom the urethra was completely obliterated in front of the scrotum; there were several urinary fistulæ, and the bladder was perforated in three different parts, but the loss of substance was closed by the intestines. An eschar was found in the bladder, exactly similar to the part of the bladder from which it had been detached. Several members of the Society thought that it was merely a false membrane, but I am convinced that it was a true eschar.—*Provincial Medical Journal*, from *Annales de Chir. Franc.* February, 1842.

